# Pig blastocyst-like structure models from embryonic stem cells

**DOI:** 10.1038/s41421-024-00693-w

**Published:** 2024-07-02

**Authors:** Jinzhu Xiang, Hanning Wang, Bingbo Shi, Jiajun Li, Dong Liu, Kaipeng Wang, Zhuangfei Wang, Qiankun Min, Chengchen Zhao, Duanqing Pei

**Affiliations:** Laboratory of Cell Fate Control, School of Life Sciences, Westlake University, Hangzhou, Zhejiang China

**Keywords:** Embryonic stem cells, Stem cells

## Abstract

Pluripotent stem cells have the potential to generate embryo models that can recapitulate developmental processes in vitro. Large animals such as pigs may also benefit from stem-cell-based embryo models for improving breeding. Here, we report the generation of blastoids from porcine embryonic stem cells (pESCs). We first develop a culture medium 4FIXY to derive pESCs. We develop a 3D two-step differentiation strategy to generate porcine blastoids from the pESCs. The resulting blastoids exhibit similar morphology, size, cell lineage composition, and single-cell transcriptome characteristics to blastocysts. These porcine blastoids survive and expand for more than two weeks in vitro under two different culture conditions. Large animal blastoids such as those derived from pESCs may enable in vitro modeling of early embryogenesis and improve livestock species’ breeding practices.

## Introduction

Generation of embryo-like structures, including blastoids, embryoids, and gastruloids from pluripotent stem cells (PSCs), provides valuable and convenient in vitro models for dissecting genetic and molecular events of embryonic development in vitro. Among them, blastoids that mimic pre-implantation blastocysts can be used to study embryogenesis and key early embryonic developmental events. It has been reported that blastoids can be generated by co-culture of mouse embryonic stem cells (ESCs) and mouse trophoblast stem cells (TSCs)^1^. Mouse extended pluripotent stem cells (EPSCs)^2–4^ and EpiSCs^5^ can form blastoids under a 3D culture system through cell lineage segregation and self-organization with limited developmental potential after post-implantation in vivo. Additionally, mouse ESCs, TSCs, and extra-embryonic endoderm stem cells (XENs) have been assembled into embryo-like structures called ETX embryoids^6,7^, and embryoids with complete gastrulation and further development into organ progenitors similar to E8.5 embryos^8,9^. Human blastoids that resemble human blastocysts in terms of morphology, cell lineage composition, and transcriptome features have been generated using naïve PSCs^10–12^ or EPSCs^13,14^ under their corresponding culture conditions. On the other hand, during the somatic reprogramming or primed-to-naïve transition, the intermediates can also be induced to form human blastoids^15,16^. Recently, the model of the post-implantation human embryo has been developed from ESCs^17–19^ and EPSCs^20^. Similarly, blastoids from non-human primates, such as cynomolgus monkeys, which capture gastrulation and early pregnancy, have been developed using naïve ESCs^21^. However, the developmental potential of human blastoids cannot be tested at the moment in vivo due to ethical constraints.

Livestock species may benefit from blastoid research. So far, only bovine blastoids have been derived by assembling EPSCs and TSCs^22^. However, blastoids from livestock species have not been generated directly from ESCs. Stable porcine ESCs (pESCs) have been derived from inner cell masses^23,24^ or epiblasts (EPI)^25,26^ under different culture conditions. Whether pESCs can be induced to form blastoids remains unclear. Here we report a novel culture condition (4FIXY) that supports the derivation of pESCs. Using these pESCs, we further develop a 3D two-step differentiation system for generating porcine blastoids. These blastoids resemble porcine blastocysts in morphology, size, cell lineage composition, and transcriptome characteristics at the single-cell level. Significantly, they can survive and expand for 18 days in two different culture conditions via prolonged in vitro culture (IVC). Given that the pig is considered a suitable surrogate for humanized organs as it shares physiological and anatomical similarities with humans^27,28^, the work presented here may aid this effort in generating implantation-competent porcine blastocysts.

## Results

### Derivation of pESCs using the 4FIXY culture medium

**Fig. 1:**
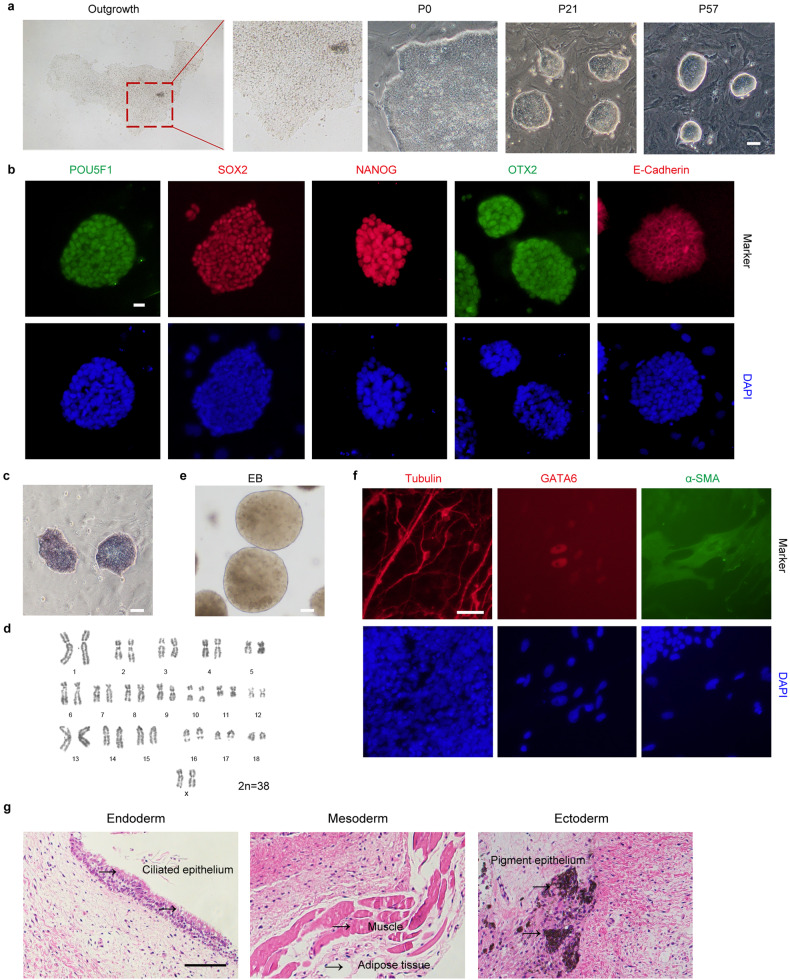
Derivation and characteristics of pESCs. **a** Representative morphology of porcine outgrowths and pESCs. Scale bar, 50 μm. **b** Representative immunofluorescent staining for pluripotency markers, including POU5F1, SOX2, NANOG, OTX2, and E-Cadherin. Scale bar, 20 μm. **c** AP staining of pESCs. Scale bar, 50 μm. **d** Karyotypes analysis of pESCs. **e** Representative morphology of EB. Scale bar, 50 μm. **f** Representative immunofluorescent staining for the ectoderm marker Tubulin, mesoderm marker α-SMA, and endoderm marker GATA6. Scale bar, 50 μm. **g** Hematoxylin and eosin staining of pESCs-derived teratomas. Scale bar, 100 μm.

Before generating porcine blastoids, we derived pESCs from parthenogenetic (PA) blastocysts using the new culture medium called 4FIXY. Based on the transcriptomic datasets from porcine early embryonic development^29^ and published culture medium^23–26^, we tested four cytokines activin A, IGF1, IL-6, and sIL-6 Receptor α, and together with some small chemical inhibitors, including WNT signal inhibitors XAV939 and IWR1, and ROCK inhibitor Y27632 to culture pESCs. The resulting pESCs can be passaged at single cells by enzymatic dissociation every 3–4 days at a ratio of 1:3 and form dense colonies with smooth and clear edges (Fig. 1a). Immunofluorescent staining demonstrates that pESCs express pluripotent markers, including POU5F1^24,25^, SOX2^24,25^, NANOG^24,25^, OTX2^25,29^, and E-Cadherin^30–32^ (Fig. 1b). During long-term culture, pESCs retain positive alkaline phosphatase (AP) staining signal and normal karyotypes (Fig. 1c, d). Furthermore, pESCs can form embryonic bodies (EBs) and differentiate into three germ layers in vitro (Fig. 1e, f). In immunodeficient mice, pESCs formed teratomas with derivatives of endoderm, mesoderm, and ectoderm germ layers in vivo (Fig. 1g). These results indicate that we have successfully derived pESCs under the 4FIXY culture condition.

We also performed bulk RNA-sequencing (RNA-seq) of pESCs and compared these cells to those in the single-cell data during early embryogenesis (oocyte–E14). The results show that pESCs are close to a cluster of E9–E11 cells, which highly express epiblast markers (*POU5F1* and *SOX2*) (Supplementary Fig. S1a). We then performed real-time quantitative PCR to examine the gene expression profile of the core, primed, and naïve pluripotent markers. The results show that core (i.e., *POU5F1*, *SOX2*, and *NANOG*) and primed (i.e., *OTX2*, *DNMT3A*, *LIN28A*, and *NODAL*) pluripotency marker genes are expressed in pESCs, while naïve pluripotent marker genes (i.e., *TBX3*, *TFCP2L1*, *STAT3*, and *KLF17*) are minimally expressed (Supplementary Fig. S1b). These data indicate that the pESCs are in the primed pluripotent state.

### Efficient generation of blastoids with pESCs

To test whether pESCs can be assembled in vitro into blastoids, we first examined their ability to differentiate into extra-embryonic lineages, including trophectoderm (TE) and hypoblast (HYPO). To this end, we established a new culture condition (hence called induced blastoid (iBlastoid) medium) to culture pESCs for 3 days. We found that EPI-, TE- and HYPO-like cells (ELCs, TLCs, and HLCs) gradually emerged (Supplementary Fig. S2a). Immunofluorescent staining showed that GATA3- and GATA6-positive cells representing TLCs and HLCs, respectively, can be identified (Supplementary Fig. S2b). GATA3- and GATA6-positive cells are both observed in the same view with SOX2-positive cells (ELCs) (Supplementary Fig. S2b). These results indicate that pESCs possess the potential to differentiate in vitro into extra-embryonic lineages, a prerequisite for blastoid generation.

We then attempted to reconstruct porcine blastoids in a 3D culture condition (Supplementary Fig. S2c). First, we prepared the iBlastoid medium, including cytokines LIF, activin A, IGF1, IL-6, and sIL-6 Receptor α based on the expression dataset of porcine early embryonic development^29^. We also used CHIR99021, SB431542, TSA, and BMP4 to support the derivation of TLCs^24,33^. Additionally, activin A, bFGF, and CHIR99021 were applied in the medium as they contribute to the differentiation of HLCs^11^. In a one-step process, pESCs were digested into single cells and directly seeded into ultra-low attachment multiple well plates at the indicated cell numbers under the iBlastoid medium. We showed that porcine blastoids containing a blastocele-like cavity, an EPI-like compartment, and an outer TE-like layer merged gradually on days 5–7 (Supplementary Fig. S2d). Immunofluorescent staining showed the expression of three major lineage markers, including EPI marker SOX2, HYPO marker GATA6, and TE marker CDX2 in these blastoids (Supplementary Fig. S2e). The proportion of SOX2-positive blastoids is between 18.4% and 34% (Supplementary Fig. S2f).

**Fig. 2:**
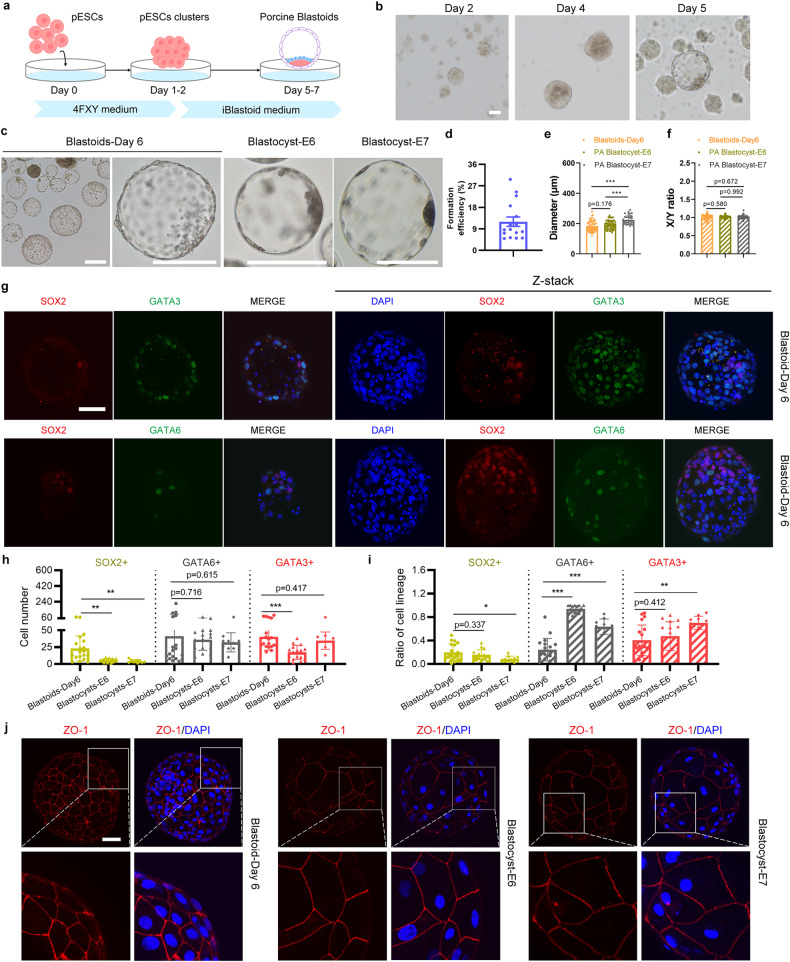
Generation of porcine blastoids using a 3D two-step strategy. **a** Schematic of porcine blastoid formation from pESCs using a 3D two-step method. **b** Representative images of cell clusters and blastoids at the indicated time points during porcine blastoid formation. Scale bar, 50 μm. **c** Representative images of porcine E6 and E7 PA blastocysts and blastoids generated from pESCs on day 6 using a 3D two-step method. Scale bars, 200 μm. **d** Formation efficiency of pESCs-derived blastoids (*n* = 16). Formation efficiency indicates the ratio of blastoids over all aggregates. **e** The diameter of porcine blastoids (*n* = 53) and porcine PA blastocysts at E6 (*n* = 50) and E7 (*n* = 33). The diameter was calculated by ImageJ. **f** The *X*/*Y* ratio of porcine blastoids (*n* = 53) and porcine PA blastocysts at E6 (*n* = 50) and E7 (*n* = 33). **g** Representative immunofluorescent staining for EPI marker SOX2, TE marker GATA3, and HYPO marker GATA6 in pESCs-derived blastoids. Scale bars, 50 μm. **h** Cell numbers of the individual lineage of per porcine blastoid (*n* = 19) and porcine PA blastocysts at E6 (*n* = 16) and E7 (*n* = 10). **i** The ratio of the individual lineage of per porcine blastoid (*n* = 19) and porcine PA blastocyst at E6 (*n* = 16) and E7 (*n* = 10). **j** Representative immunofluorescent staining for tight junction marker ZO-1 in porcine blastoid and porcine PA blastocysts at E6 and E7. Scale bar, 50 μm. *P < 0.05; **P < 0.01; ***P < 0.001. The *P* values were calculated using unpaired *t*-tests.

To improve the efficiency of porcine blastoid generation, we modified and developed a more robust 3D two-step method (Fig. 2a; Supplementary Fig. S2f). In this two-step induction process, pESCs dissociated into single cells were seeded into ultra-low attachment multiple well plates in 4FXY medium (4FIXY minus IWR1) to form cell clusters on days 1–2; cell clusters were then maintained in iBlastoid medium for the following 3–5 days (Fig. 2a). The proportion of SOX2-positive blastoids ranges from 25.9% to 55% (Supplementary Fig. S2f). In iBlastoid medium, cell clusters gradually formed the blastocele-like cavity, EPI-like cells, and TE-like layers (Fig. 2b). In vitro-derived blastoids on day 6 are morphologically similar to porcine PA blastocysts at E6 and E7 (Fig. 2c). The efficiency of porcine blastoid formation ranges from 4.95% to 29.68% (Fig. 2d). Additionally, the diameters of porcine blastoids (mean, 183 μm) are similar to porcine PA blastocysts at E6 (mean, 192 μm), which are both smaller than E7 blastocysts (mean, 224 μm) (Fig. 2e). The *X*/*Y* ratio of porcine blastoids is approximately equal to 1, which indicates the round shape of blastoids and is equivalent to that of porcine blastocysts at E6 and E7 (Fig. 2f). Taken together, we show that porcine blastoids can be generated efficiently from pESCs in a 3D system.

### Porcine blastoids exhibit characteristic features of blastocysts

To explore whether reconstructed porcine blastoids contained the characteristic three lineages of blastocysts, we performed immunofluorescent staining to detect markers for EPI (SOX2)^25,29^, HYPO (GATA6)^25,29^, and TE (GATA3)^25,29^ and showed that porcine blastoids contain these cells (Fig. 2g). We also confirmed additional markers for EPI (POU5F1)^25,29^, HYPO (GATA4)^25,29^, and TE (CDX2)^25,29^ in porcine blastoids (Supplementary Fig. S3). Notably, EPI (SOX2, POU5F1) and HYPO (GATA6, GATA4) markers were mainly expressed in the inner cells, and TE markers GATA3 and CDX2 were expressed in the outer cells (Fig. 2g; Supplementary Fig. S3). Then, we calculated the cell numbers and ratio of these three cell lineages in porcine blastoids. The blastoids contain more cells than E6 and E7 PA blastocysts. Compared to E6 blastocysts, these porcine blastoids contain more ELCs and TLCs, while the proportions of ELCs and TLCs are comparable to E6 blastocysts (Fig. 2h, i). The numbers of HLCs are similar, while the ratio of HLCs in blastoids is lower than that in E6 blastocysts (Fig. 2h, i). Compared to E7 blastocysts, cell numbers and proportions of ELCs are both higher. HLCs and TLCs numbers are similar while their ratio is lower than that in E7 blastocysts (Fig. 2h, i). Intercellular tight junctions between the TLCs of blastoids can be detected by immunofluorescent staining of ZO-1 (Fig. 2j). Taken together, our data demonstrate that the pESCs-derived blastoids display characteristics similar to porcine blastocysts.

### Single-cell transcriptomics of porcine blastoids

**Fig. 3:**
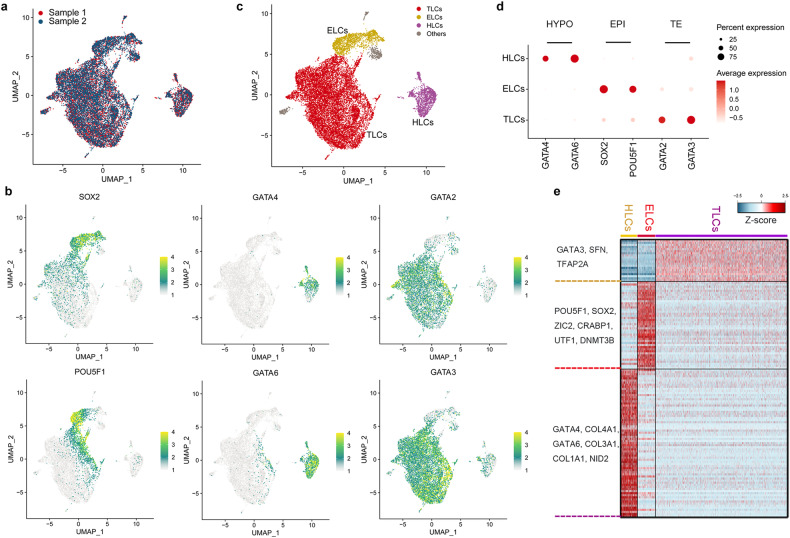
Single-cell transcriptional profiling of porcine blastoids. **a** UMAP representation of two sample distributions for porcine blastoids. **b** UMAP showing expression of EPI markers (*SOX2*, *POU5F1*), HYPO markers (*GATA4*, *GATA6*), and TE markers (*GATA2*, *GATA3*). **c** Cells derived from porcine blastoids are colored by origin: ELCs (yellow), HLCs (purple), and TLCs (red). **d** Dot plot indicating EPI markers (*SOX2*, *POU5F1*), HYPO markers (*GATA4*, *GATA6*), and TE markers (*GATA2*, *GATA3*). **e** Heatmap representing the expression of cell lineage-specific genes in ELCs, HLCs, and TLCs.

To determine the transcriptional profiles of porcine blastoid-derived cells, we performed single-cell RNA sequencing (scRNA-seq) from two samples (Fig. 3a). Uniform manifold approximation and projection (UMAP) analysis revealed that porcine blastoid-derived cells were divided into 13 clusters (Supplementary Fig. S4a). We identified ELCs, HLCs, and TLCs using known signature markers such as *SOX2* and *POU5F1* for ELCs; *GATA6* and *GATA4* for HLCs; and *GATA3* and *GATA2* for TLCs (Fig. 3b, c; Supplementary Fig. S4b). Clusters were assigned to the most likely cell type based on two key criteria: the percentages of cells expressing the marker genes within the cluster and the scaled average expression level. Cell clusters revealed specific expressions of different lineage markers. Cluster 5 expresses *SOX2* and *POU5F1* and represents ELCs; clusters 7 and 10 express *GATA6* and *GATA4* and are regarded as HLCs; seven clusters (0–4, 6, 12) express *GATA3* and *GATA2* and are annotated as TLCs (Fig. 3b; Supplementary Fig. S4a). For clusters 8, 9, and 11, where the percentages were < 50% and the scaled average expression level was below 0.1 for all the marker genes, and determining the most likely cell type was challenging. Thus, these clusters were excluded. Each of the porcine blastoid lineages showed distinct gene expression patterns that are similar to their corresponding lineage in porcine embryos (Fig. 3d, e; Supplementary Fig. S4c). For instance, known EPI genes^25,29^ (*DNMT3B*, *ETV5*, *ZIC2*) are specific to the ELC cluster. HYPO gene *NID2*^25,29^ is specific to the HLC cluster; another HYPO gene *COL4A1*^25,29^, is highly expressed in the HLC cluster. Known TE genes^25,29^ (*KRT8*, *SFN*) are most highly expressed in TLC clusters (Supplementary Fig. S4d, e). We then assessed the resolving power by clustering with different parameter combinations. Except for a few combinations where the resolution is too low to distinguish some small cell populations, most parameter combinations consistently produce results in defining cell types (Supplementary Fig. S5). These results confirm the presence of EPI-, HYPO-, and TE-like lineage cells in the porcine blastoids.

**Fig. 4:**
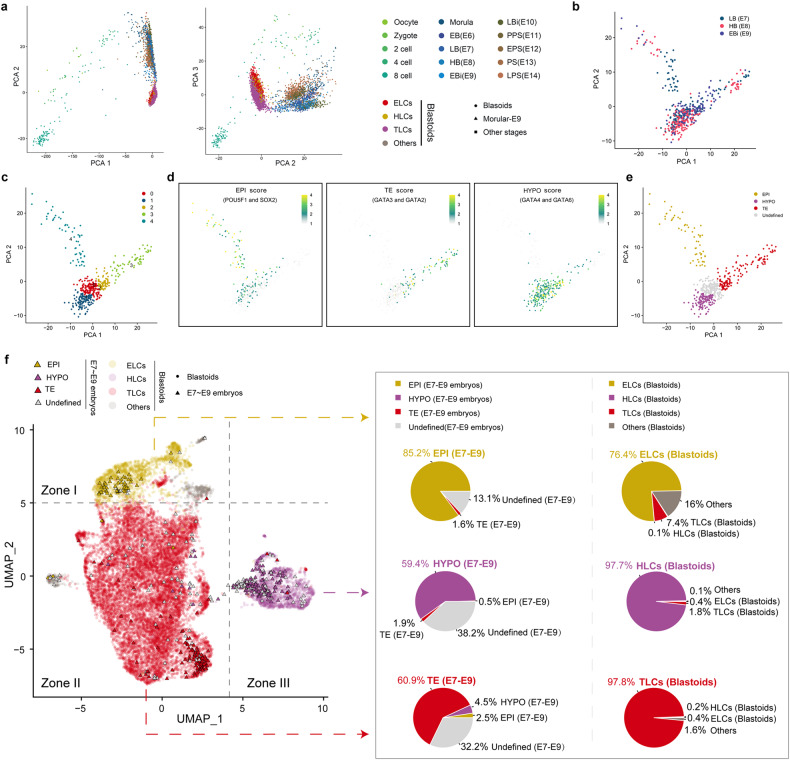
Integrated scRNA-seq analysis of porcine blastoids and embryos. **a** PCA of porcine blastoids and embryos^25^. EB (early blastula, E6), LB (late blastula, E7), HB (hatched blastula, E8), EBi (early bilaminar embryo, E9), LBi (late bilaminar embryo, E10), PPS (pre-primitive streak embryo, E11), EPS (early primitive streak embryo, E12), PS (primitive streak embryo, E13), and LPS (late primitive streak embryo, E14). **b** PCA of LB, HB, and EBi. **c** PCA analysis showing that porcine embryos^25^ at E7–E9 included five clusters. **d** Expression scores of EPI (*POU5F1* and *SOX2*), TE (*GATA2* and *GATA3*), and HYPO (*GATA4* and *GATA6*). **e** Cells derived from porcine embryos are colored by origin: EPI (yellow), HYPO (purple), and TE (red). **f** UMAP plot of porcine blastoid cells integrated with published porcine embryo cells^25^ and a pie chart showing the percentages of three lineage cells. The percentages 85.2%/59.4%/60.9% are calculated by dividing the number of EPI/HYPO/TE lineage cells by the number of embryo-derived cells in zone I/II/III, respectively. Similarly, the percentages 76.4%/97.7%/97.8% are calculated by dividing the number of ELCs/HLCs/TLCs by the number of blastoid-derived cells in zone I/II/III, respectively.

Next, we asked whether the porcine blastoids derived from pESCs resemble porcine blastocysts at the transcriptome level. We carried out integrated analysis with single-cell transcriptomes derived from porcine oocytes and embryos at different stages^25^. Principal component analysis (PCA) revealed that blastoid-derived cells can be mapped close to blastocyst cells (Fig. 4a). To further evaluate the similarities between lineage cells from blastoids and blastocysts, we employed two comparison methods for integrated analysis, which can unbiasedly and quantitatively benchmark the blastoid-derived cells. In pig early embryonic development, the first lineage segregation is initiated at E5; the second lineage segregation begins at E6 and ends at the E7 stage^25,29^; EPI then transits to ectoderm from E10 to E11^25^, demonstrating that three lineage cells, including EPI, TE, and HYPO, are well segregated at E7, E8, and E9. Therefore, to investigate the correlation between blastoids and blastocyst-derived cells for all three lineages (ELCs with EPI, TLCs with TE, and HLCs with HYPO), single-cell transcriptomes of porcine embryos at E7, E8, and E9 were chosen to perform integrated analysis (Fig. 4b). We identified EPI, HYPO, and TE using markers *SOX2* and *POU5F1* for EPI; *GATA6* and *GATA4* for HYPO; and *GATA3* and *GATA2* for TE (Fig. 4c–e; Supplementary Fig. S6a). We then performed UMAP analysis and showed that ELCs, TLCs, and HLCs in blastoids overlap with their EPI, TE, and HYPO counterparts from blastocysts, respectively (Fig. 4f). Additionally, we utilized scPred^34^, an accurate supervised cell-type classification method to classify blastoid-derived cell types based on E7–E9 embryo datasets. Blastoid-derived cells were projected onto the E7–E9 embryo datasets that were classified into lineages of EPI, HYPO, TE, and undefined clusters. scPred predicted that 91%, 85%, and 54% of blastoid-derived cells possessed EPI, HYPO, and TE lineage characteristics, respectively, in their corresponding lineage group (Supplementary Fig. S6b). Overall, these results suggest that pESCs-derived blastoids possess the three characteristic lineages and, thus, very similar transcriptomic features to in vivo physiological embryos, i.e., blastocysts.

### Derivation of stem cells from porcine blastoids

**Fig. 5:**
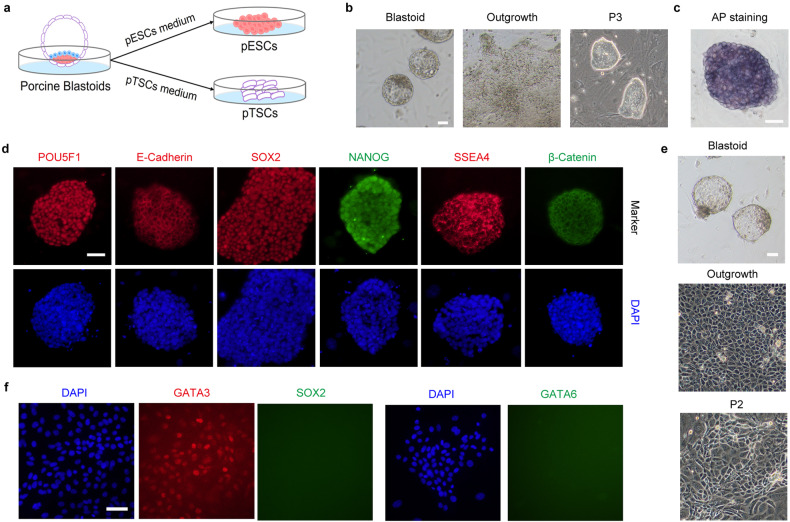
Derivation of stem cells from porcine blastoids. **a** Illustration of the establishment of pESCs and pTSCs from blastoids. **b** Representative morphology of outgrowth and pESCs derived from blastoids. Scale bar, 50 μm. **c** AP staining of pESCs derived from blastoids. Scale bar, 50 μm. **d** Representative immunofluorescent staining for pluripotent markers POU5F1, SOX2, NANOG, SSEA4, β-Catenin, and E-Cadherin in blastoids-derived pESCs. Scale bar, 50 μm. **e** Representative morphology of outgrowth and pTSCs derived from blastoids. Scale bar, 50 μm. **f** Representative immunofluorescent staining for GATA3, SOX2, and GATA6 in TSCs. Scale bar, 50 μm.

We then ask whether we can derive stem cells from these blastoids as we did from blastocysts, as shown in Fig. 1a. To this end, we show that porcine blastoids support de novo derivation of ESCs in the same culture condition and TSCs in the indicated medium (Fig. 5a). The outgrowths from blastoids can be formed and pESCs generated with compact colonies (Fig. 5b) that are positive for AP staining and express pluripotent markers, including POU5F1, E-Cadherin, SOX2, NANOG, SSEA4, and β-Catenin (Fig. 5c, d). Using a TSC medium, porcine TSCs (pTSCs) can also be established and express TE marker GATA3 (Fig. 5e, f). Collectively, these results suggest that these blastoids are amenable to stem cell derivation as blastocysts.

### Prolonged culture of porcine blastoids

**Fig. 6:**
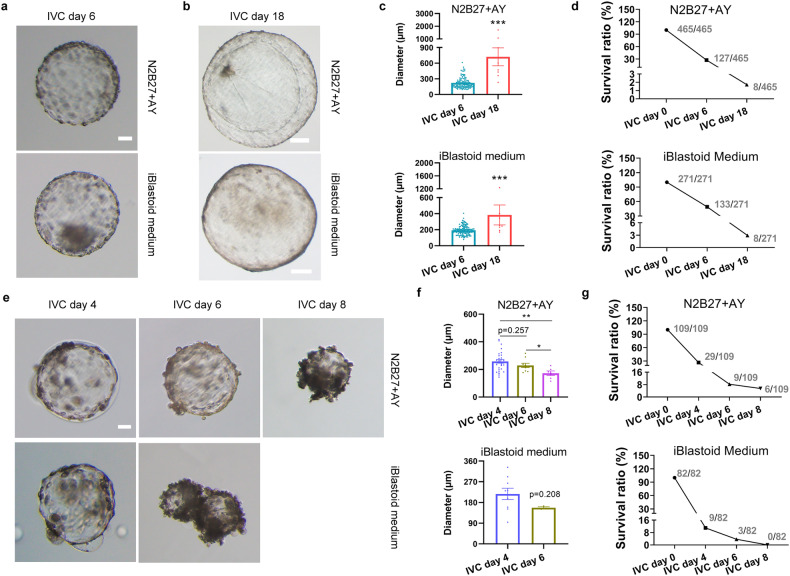
In vitro cultured porcine blastoids. **a** Representative morphology of in vitro cultured blastoids on day 6. Scale bar, 50 μm. **b** Representative morphology of in vitro cultured blastoids on day 18. Scale bars, 200 μm. **c** The diameters of in vitro cultured blastoids. The diameter was calculated by ImageJ. ***P < 0.001. The *P* values were calculated using unpaired *t*-tests. **d** The survival ratio of in vitro cultured blastoids. **e** Representative morphology of in vitro cultured porcine PA blastocysts on days 4, 6, and 8. Scale bar, 50 μm. **f** The diameters of in vitro cultured porcine PA blastocysts. The diameter was calculated by ImageJ. *P < 0.05; ** *P* < 0.01. The *P* values were calculated using unpaired *t*-tests. **g** The survival ratio of in vitro cultured porcine PA blastocysts.

We then explore the possibility of these blastoids growing and developing in vitro. To the best of our knowledge, the IVC approach of porcine embryos has not been established. According to IVC systems of bovine and ovine embryos^22,35^, we modified these culture conditions and developed N2B27 + AY medium for blastoid culture. The iBlastoid medium was also used for in vitro prolonged culture. Porcine blastoids on day 6 (denoted as porcine blastoid IVC day 0) with EPI-like cells and apparent cavity were chosen for in vitro prolonged culture. When IVC of porcine blastoids continues to day 18, we can see cavities of blastoids that continue to survive and expand, accompanied by an increased size of blastoids (Fig. 6a, b). The diameters of porcine blastoids range from 234 to 1707 μm (mean, 722 μm) in N2B27 + AY medium, compared to 171 to 1234 μm (mean, 384 μm) in iBlastoid medium on day 18 (Fig. 6c). The survival ratios of blastoids are 27.31% and 49.07% in N2B27 + AY and iBlastoid medium, respectively, on day 6, and decline to 1.72% and 2.96%, respectively, on day 18 (Fig. 6d). As controls, porcine PA blastocysts grow for about 6 and 8 days in N2B27 + AY and iBlastoid medium, respectively, before becoming smaller as IVC days progress (Fig. 6e–g), indicating that the culture conditions are not yet to be optimal for pig embryos.

Generally, mouse and human embryos undergo a series of developmental stages, including pre-implantation, implantation, and gastrulation. Implantation starts before gastrulation^36^. However, in porcine early embryo development, conceptus TE first initiates elongation and rearrangement on days 11–12 of gestation. Gastrulation then starts, followed by implantation, which is very different in mice and humans^36^. The elongation is characterized by trophoblast morphological rearrangement and transition through four distinct morphological stages, including spherical, ovoid, tubular, and filamentous forms^37^. In this study, IVC blastoids exhibit spherical shapes (Fig. 6a, b, e), indicating that the developmental stage occurs on days 8–10 of gestation before elongation. As embryos on days 8–10 of gestation contain three lineage cells (EPI, HYPO, and TE), we performed immunofluorescent staining of the three lineage markers (SOX2 for EPI, GATA6 for HYPO, and GATA3 for TE) in IVC blastoids. The results show that IVC blastoids on day 18 contain SOX2, GATA3, and GATA6-positive cells (Supplementary Fig. S7). Overall, these results indicate that the blastoids have the potential to survive and expand in vitro prolonged culture.

## Discussion

Although the generation of stable porcine PSCs isolated from embryos or reprogrammed from somatic cells has been attempted since the 1990s, it lagged behind mouse and human PSCs. Several published PSCs exhibit common self-renewal requirements^23,25,26^ and can be used to generate cloned piglets by gene editing^25^. Porcine EPSCs possess embryonic and extra-embryonic developmental potency^24^. In our study, we applied several cytokines (activin A, IGF1, IL-6, and sIL-6 Receptor α) and chemical inhibitors (XAV939, IWR1, and Y27632) to derive pESCs from PA blastocysts that share common pluripotency and differential potency in vitro and in vivo. The pESCs match closely to E9–E11 cells and express primed pluripotent genes, whose state is similar to the published pESCs^23–26^. As we all know, porcine PA^23,24^, in vitro fertilization (IVF)^23^, and in vivo^25^ embryos were generally used to generate ESCs. The efficiency of the derivation of ESCs from PA and IVF blastocysts was similar^23^. Therefore, we think our methods and culture conditions may apply to pESC derivation from IVF and in vivo embryos.

Recent advances have demonstrated that mouse, human, and monkey ESCs can be induced to generate blastoids^1,13,21^. These blastoids mimic the blastocyst morphology, cell lineage composition, and transcriptome. However, their developmental capacities remain limited. To date, ESCs from livestock species, such as pig, bovine, and sheep, have not been induced to form blastoids. In this work, we first developed a condition, i.e., the 4FIXY medium, to support the derivation of pESCs. More critically, we show that the resulting pESCs can be assembled in vitro into blastocyst-like structures, which we call blastoids.

**Fig. 7:**
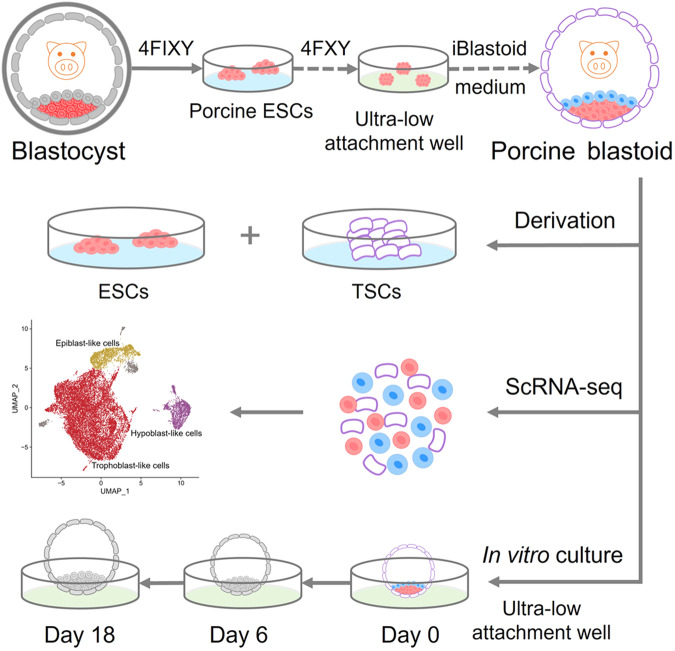
Schematic illustrating the major findings of this study.

There are some differences between porcine blastoids and blastocysts, such as cell numbers of lineages and proportions of the HYPO lineage. Blastoid modeling technology is at very early stages that requires additional investigation and optimization, particularly in developmental signaling, cell fate determination, and lineage segregation. Nevertheless, we show that these porcine blastoids recapitulate morphogenetic events during early embryo development and provide a valuable in vitro model to explore porcine embryogenesis. Future work may help solve the above-mentioned differences as well as develop more optimized conditions for prolonged culture in vitro. Future strategies are needed to improve porcine blastoid qualities and its developmental capacity. By analyzing this 3D induction system, one may identify additional means to improve the process (Fig. 7). While we have not implanted these blastoids into surrogates, we intend to secure the necessary approval and resources to do so in the near future.

## Materials and methods

### Animal treatment and ethics statements

All animal experiments were approved by the Animal Care and Use Committee of Westlake University.

### Establishment of pESCs

Porcine oocytes with multiple layers of cumulus cells were collected from the follicle, matured in vitro for 42–46 h, and treated with hyaluronidase to remove cumulus cells. Oocytes were then exposed to a pulse of 60 V for 30 μs in the activation medium followed by incubation in PZM-3 medium^38^. For the derivation of pESCs, the whole PA blastocysts were digested with 0.5% pronase (Sigma-Aldrich, P8811) to remove zona pellucida and then cultured in the 4FIXY or 4FXY medium plus 5% FBS on feeder cells until blastocysts were adherent. The outgrowths were dissociated with TrypLE^TM^ Select and reseeded onto mitomycin C-treated mouse embryonic fibroblast feeder cells in 4FIXY medium. The cells formed well-fined colonies about 3 days later. Cells were cultured at 38.5 °C, 5% O_2_, and 5% CO_2_.

The 4FIXY medium was composed of 1:1 (v/v) mix of Neurobasal medium (Gibco, 21103049) and DMEM/F12 (Gibco, 10565018) supplemented with N2 (Gibco, 17502048) and B27 (Gibco, 17504044) supplements, NEAA (Gibco, 11140050), GlutaMAX (Gibco, 35050061), penicillin/streptomycin (Gibco, 15140122), 5% KOSR, 100 μM 2-mercaptoethanol (Sigma-Aldrich, M3148), 0.15% FBS (Gibco, 10099141C), 20 ng/mL human IL-6 (Peprotech, AF-200-06), 20 ng/mL human sIL-6 Receptor α (Peprotech, 200-06RC), 20 ng/mL activin A (Peprotech, 120-14-1000), 50 ng/mL human IGF1 (MCE, HY-P7018), 2.5 μM XAV939 (Selleck, S1108), 2.5 μM IWR1 (Selleck, S7086), 50 µg/mL 2-Phospho-L-ascorbic acid trisodium salt (Sigma-Aldrich, 49752) and 5 μM Y-27632 (TargetMol, T1725). The 4FXY medium was the 4FIXY medium minus IWR1.

### Culturing pESCs

The pESCs were maintained on mitomycin C-treated mouse embryonic fibroblast feeder cells and enzymatically passaged every 3–4 days. The cells were dissociated as single cells by TrypLE^TM^ Select (38.5 °C, 5 min), centrifuged (250× *g*, 5 min), resuspended, and seeded in 4FIXY medium at a ratio of 1:3. Cells were cultured at 38.5 °C, 5% O_2_, and 5% CO_2_.

### EB formation

Pre-differentiated ESCs were detached using TrypLE^TM^ Select (38.5 °C, 5 min) and plated to ultra-low attachment multiple well plates in EB formation medium for 7 days. The EBs were attached on the gelatin-coated plates in DMEM (HyClone, SH30022.01B) supplemented with 15% FBS and 5 μM Y-27632 for 3 days, cultured in DMEM plus 15% FBS for 4 days, and collected for analysis. Cells were cultured at 38.5 °C, 5% O_2_, and 5% CO_2_.

The EB formation medium was composed of 1:1 (v/v) mix of Neurobasal medium (Gibco) and DMEM/F12 (Gibco) supplemented with N2 (Gibco) and B27 (Gibco) supplements, NEAA (Gibco), GlutaMAX (Gibco), penicillin/streptomycin (Gibco), 5% KOSR, 100 μM 2-mercaptoethanol (Sigma-Aldrich) and 5 μM Y-27632 (TargetMol).

### Generation of porcine blastoids

In the one-step process, pESCs were digested with TrypLE^TM^ Select into single cells at 38.5 °C for 5 min. Cells were collected and resuspended in iBlastoid medium. About 40,000 single cells per well were directly seeded into ultra-low attachment multiple well plates under iBlastoid medium for 5–7 days. In the two-step induction process, pESCs were dissociated with TrypLE^TM^ Select into single cells at 38.5 °C for 5 min. Cells were collected and resuspended in 4FXY medium. About 40,000 single cells per well were seeded into ultra-low attachment multiple well plates in 4FXY medium for 1–2 days. Then, cell clusters formed from pESCs were cultured in iBlastoid medium for 3–5 days. All generated porcine blastoids were manually isolated using a mouth pipette under a stereomicroscope for downstream experiments. Blastoids were cultured at 38.5 °C, 20% O_2_, and 5% CO_2_.

The iBlastoid medium was composed of 1:1 (v/v) mix of Neurobasal (Gibco) medium and DMEM/F12 (Gibco) supplemented with N2 (Gibco), and B27 (Gibco) supplements, NEAA (Gibco), GlutaMAX (Gibco), penicillin/streptomycin (Gibco), 5% KOSR, 0.075% FBS, 100 μM 2-mercaptoethanol (Sigma-Aldrich), 50 µg/mL 2-Phospho-l-ascorbic acid trisodium salt (Sigma-Aldrich), 10 ng/mL human IL-6 (Peprotech), 10 ng/mL human sIL-6 Receptor α (Peprotech), 10 ng/mL activin A (Peprotech), 5 ng/mL human LIF (Peprotech, 300-025), 50 ng/mL human IGF1, 5 ng/mL BMP4 (R&D, 314-BP), 10 ng/mL bFGF (Peprotech, 100-18B), 1.5 μM CHIR99021 (Tocris, 4423), 2.5 μM XAV939 (Selleck), 1 μM SB431542 (Selleck, S1067), 7.5 μM Y-27632 (TargetMol), 2.5 nM DZNep (Selleck, S7120), and 2.5 nM TSA (Solarbio, IT1250).

### Derivation of stem cell lines from porcine blastoids

Individual porcine blastoids were transferred onto mitomycin C-treated mouse embryonic fibroblast feeder cells in a four-well plate and cultured in 4FIXY medium (for pESCs) and TSC medium (for pTSCs). Outgrowths observed within 7 days were dissociated with TrypLE^TM^ Select and passaged onto newly mitomycin C-treated mouse embryonic fibroblast feeder cells in their corresponding medium. 5% FBS was added to the medium until blastoids were adherent. Cells were cultured at 38.5 °C, 5% O_2,_ and 5% CO_2_.

TSC medium was modified according to the previous report^33^, which was composed of 1:1 (v/v) mix of Neurobasal medium (Gibco) and DMEM/F12 supplemented with N2 (Gibco) and B27 (Gibco) supplements, NEAA (Gibco), GlutaMAX (Gibco), penicillin/streptomycin (Gibco), 100 μM 2-mercaptoethanol (Sigma-Aldrich), 50 µg/mL 2-Phospho-l-ascorbic acid trisodium salt, 50 ng/mL human EGF (Peprotech, AF-100-15), 1 μM SB431542, 2 μM CHIR99021, 0.8 mM VPA (Solarbio, IV0010), 10 μM Y-27632 (TargetMol) and 0.5 μM A83-01 (STEMCELL, 72022).

### In vitro prolonged culture of porcine blastoids

Porcine blastoids were manually isolated using a mouth pipette and transferred into new ultra-low attachment multiple well plates containing iBlastoid medium or N2B27 + AY medium. Blastoids were cultured at 38.5 °C, 20% O_2_, and 5% CO_2_.

N2B27 + AY medium was modified according to the IVC medium of bovine and ovine embryos^22,35^. N2B27 + AY medium was composed of a 1:1 (v/v) mix of Neurobasal medium and DMEM/F12 supplemented with N2 and B27 supplements, NEAA, GlutaMAX, penicillin/streptomycin, 5% KOSR, 100 μM 2-mercaptoethanol, 20 ng/mL activin A, and 10 μM Y-27632.

### Real-time quantitative PCR

Cells were collected, and total RNA was extracted using TRIZOL (Invitrogen) according to the manuscript’s instructions. Reverse transcription was performed using HiScript II Q RT SuperMix (Vazyme, R222). Quantitative PCR reactions were performed using SYBR qPCR Master Mix (Vazyme, Q711) and run on CFX96 Touch Real-Time PCR Detection System (BIO-RAD). Relative expression values were normalized to EF1α. Primers used for real-time quantitative PCR were shown in Supplementary Table S1.

### Immunofluorescent staining

Cells, blastoids, and blastocysts were fixed with 4% paraformaldehyde (PFA) for about 30 min at room temperature, washed in PBS for 5 min, and permeabilized with 0.2% Triton X-100 in PBS for 30 min. For surface marker staining, samples were not permeabilized. Samples were then blocked with a blocking buffer at room temperature for 40 min. Primary antibodies were diluted in a primary antibody dilution buffer. Blastoids/blastocysts in 96 wells or cells were incubated with primary antibodies overnight at 4 °C. Samples were washed three times for 15 min with PBS and incubated with fluorescent-dye conjugated secondary antibodies diluted in secondary antibody dilution buffer for 1 h at room temperature. Samples were washed three times with PBS. Finally, samples were counterstained with DAPI at room temperature for 3 min. Blastoids were imaged on 8 well μ-slides (ibidi). Blastocysts were imaged in a glass slide under a coverslip.

### Karyotype analysis

Before karyotype analysis, pESCs were treated with 4 μg/mL colchicine in culture medium for 2 h. The cells were digested, centrifuged, resuspended with 0.075 M KCl hypotonic solution, and incubated at 37 °C for 15 min. After that, pESCs were fixed with a precooled 3:1 (v/v) mix of methanol and acetic acid, and this step was repeated three times. The resuspended pESCs were dropped on precooled slides, dried, and stained with Giemsa.

### Teratoma formation

About 0.5–1 × 10^7^ cells of pESCs were digested and collected by centrifugation (250 × *g*, 5 min) and subcutaneously injected into the dorsal flank of NCG mice. After about 10 weeks, teratomas were collected and processed for sectioning.

### Hematoxylin and eosin staining

Teratomas were collected and fixed with 4% PFA for over 24 h. Teratoma tissues were dehydrated with gradient alcohol, transferred into xylene, embedded in paraffin, and sectioned into serial cross-sections of a 5-μm thickness. The cross-sections were deparaffinized in xylene, rehydrated with a gradient of ethanol, and stained with hematoxylin and eosin.

### Bulk RNA-seq and analysis

A total amount of 1 µg RNA was used as input material. Sequencing libraries were generated using NEBNext® UltraTM RNA Library Prep Kit for Illumina^®^, and index codes were added to attribute sequences to each sample. Library quality was assessed on the Agilent Bioanalyzer 2100 system. The library preparations were sequenced on an Illumina NovaSeq platform (Illumina, USA), and 150 bp paired-end reads were generated. The RNA-seq reads were trimmed using Trim Galore (v0.6.4) and then mapped to the susScr11 reference genome with HISAT2 (v2.2.1), and StringTie (v2.2.1) was used to quantify the transcription level of each gene in each sample into FPKM (Fragments per kilobase of exon per million mapped reads).

### scRNA-seq

Porcine blastoids with EPI-like and TE-like compartments and cavities were picked up manually by mouth pipette and dissociated into single cells by 0.25% trypsin–EDTA at 37 °C for 10–20 min. The dissociation was terminated by 15% FBS, and samples were centrifuged at 500× *g* for 5 min. Cell pellets were resuspended in DMEM/F12 and filtered through a 40-μm cell strainer. Single-cell suspensions were carried out with 10x Genomics Chromium Single Cell Kit for Single-cell RNA-seq.

### scRNA-seq data collection

The porcine embryo dataset included CRA003960^25^.

### Single-cell RNA-seq data analysis

The scRNA-seq reads were mapped to the susScr11 reference genome using 10x Genomics Cell Ranger (v7.1.0)^39^ after quality control by Dr.seq2 (v2.2.1)^40^. Following that, Seurat (v4.3.0)^41^ was used to integrate samples using the transcription level of each gene in each sample as input. We first identified the nearest neighbors of each single cell, followed by unbiased clustering using a graph-based approach with the Louvain algorithm on the two dimensions reduced by UMAP analysis. Subsequently, clusters were assigned to the most likely cell type based on two key criteria: the percentages of cells expressing the marker genes within each cluster and the scaled average expression level. Clusters in which over 50% of cells expressing marker genes for a specific lineage and an average expression level exceeding 0.1 were regarded as specific lineage-like cells. This was the case for most clusters. For clusters that met these criteria for multiple lineage-specific markers, they were regarded as the lineage-like cells with the highest expression level. Clusters that did not meet the criteria for any lineages were excluded. Dimensional reduction and clustering were performed using Seurat function RunUMAP(), FindNeighbors(), and FindCluster() (with parameters k.param = 30 and resolution = 0.15) after normalization. The specific expressed genes with an average log fold change > 0.8 and a differential expressed percentage > 30% for each cell type were found using the FindAllMarkers() function. The scaled average expression value of given markers for each lineage was calculated as the expression score (EPI: *POU5F1* and *SOX2*, TE: *GATA3* and *GATA2*, HYPO: *GATA6* and *GATA4*). Seurat (v4.3.0) was used to assess cluster similarities between blastoids and E7–E9 embryos^25^. scPred^34^ was used to classify cell types of porcine blastoid-derived cells based on the E7–E9 embryo dataset. scPred utilized an accurate supervised approach combining unbiased feature selection and a machine-learning probability-based prediction method to classify cell types. In the training step, the gene expression matrix undergoes eigen decomposition to extract orthonormal linear combinations of gene expression values. Then, informative principal components (PCs) are selected using a two-tailed Wilcoxon signed-rank test for each cell class distribution. The cell-PC matrix is divided into *k* groups; the first group is used as a testing set for cross-validation, while the remaining *k*−1 groups are used to train a classification model (SVM). The best-performing model is selected based on its prediction accuracy. In the prediction step, gene expression values from each cell are projected onto the trained principal component basis, using these PCs to predict cell class probabilities with the model. Specifically, in our study, we trained the scPred model using scRNA-seq data of published porcine early embryos^25^. Based on early embryo scRNA-seq data, cell lineages with their respective characteristics, including EPI, HYPO, and TE, are identified. Other cells with multiple characteristics are considered as “Undefined”. Applying this prediction model to scRNA-seq data of porcine blastoids resulted in the classification of blastoid-derived cells into four cell classes (EPI lineage, HYPO lineage, TE lineage, and Undefined).

### Statistical analysis

The *P* values were calculated using unpaired *t*-tests and shown in the related figures. *P* < 0.05 is considered significant.

### Supplementary information

Supplementary Information

## Data Availability

The RNA-seq and scRNA-seq data have been deposited in the Gene Expression Omnibus database under the accession number GSE242553. Further information of the current study is available from the corresponding author on reasonable request.
