# Supplementary material for: Pig blastocyst-like structure models from embryonic stem cells
Source: Cell Discov. 2024 Jul 2;10:72. doi: 10.1038/s41421-024-00693-w (PMC11219778; doi:10.1038/s41421-024-00693-w)

Supplemental Information

Pig Blastocyst-like Structure Models from Embryonic Stem Cells

Jinzhu Xiang[1,2], Hanning Wang[1,2], Bingbo Shi[1], Jiajun Li[1], Dong Liu[1], Kaipeng Wang[1], Zhuangfei Wang[1], Qiankun Min[1], Chengchen Zhao[1*], Duanqing Pei[1*]

[1] Laboratory of Cell Fate Control, School of Life Sciences, Westlake University, Hangzhou, China 310030
[2] These authors contributed equally: Jinzhu Xiang and Hanning Wang
* E-mail: zhaochengchen@westlake.edu.cn; peiduanqing@westlake.edu.cn

Supplementary Fig. S1 The pESCs exhibited the primed pluripotent state. a Integrated analysis of pESCs and published embryo data[1]. EB (early blastula, E6), LB (late blastula, E7), HB (hatched blastula, E8), EBi (early bilaminar embryo, E9), LBi (late bilaminar embryo, E10), PPS (pre-primitive streak embryo, E11), EPS (early primitive streak embryo, E12), PS (primitive streak embryo, E13), and LPS (late primitive streak embryo, E14). **b** Real-time quantitative PCR data of pluripotent markers in pESCs. Core pluripotent genes included *POU5F1*, *SOX2*, and *NANOG*; Primed genes included *DNMT3A*, *LIN28A*, *OTX2*, and *NODAL*; Naïve genes included *KLF17*, *TBX3*, *STAT3*, and *TFCP2L1*.

Supplementary Fig. S2 Generation and optimization of porcine blastoids. a Representative image of ELCs, TLCs, and HLCs in iBlastoid medium. Scale bar, 50 μm. **b** Representative immunofluorescent staining for GATA3, SOX2, and GATA6 showing the differentiation of pESCs into TE, EPI, and HYPO lineages at indicated medium. Scale bar, 50 μm. **c** Schematic depiction of porcine blastoid induction from pESCs using the one-step method. **d** Representative images of porcine blastoids generated using the one-step method. Scale bar, 200 μm. **e** Representative immunofluorescent staining for markers of EPI (SOX2), TE (CDX2), and HYPO (GATA6) in reconstructed blastoids. Scale bar, 50 μm. **f** Comparison of generating blastoids using one-step and two-step methods.

Supplementary Fig. S3 Expression of three lineage markers of porcine blastoids and blastocysts. a Immunofluorescent staining for EPI markers (SOX2, POU5F1), TE markers (GATA3, CDX2), and HYPO marker (GATA4) in pESCs-derived blastoids. Scale bars, 50 μm. **b** Immunofluorescent staining for markers of EPI (SOX2), TE (GATA3), and HYPO (GATA6) in pESCs-derived blastoids. Scale bars, 50 μm. **c** Immunofluorescent staining for markers of EPI (SOX2), TE (GATA3), and HYPO (GATA6) in porcine blastocysts at E6 and E7. Scale bars, 50 μm.

Supplementary Fig. S4 A single-cell landscape of pESCs-derived blastoids. a UMAP analysis showing that porcine blastoids included thirteen clusters. **b** Identification of cell lineages based on lineage markers. **c** Expression score of ELCs, TLCs, and HLCs. **d** UMAP showing expression of known EPI genes (*ETV5*, *DNMT3B*), HYPO genes (*NID2*, *COL4A1*), and TE genes (*SFN*, *KRT8*). **e** Dot plot indicating the selected EPI, HYPO, and TE lineage genes.

Supplementary Fig. S5 A single-cell landscape of blastoids under different parameters. a The flow chart

detailing the parameter selection for the definition of ELC, HLC, and TLC populations. **b** Heatmap showing the Jaccard index of various parameters measuring the consistency of cell type assignments. **c** UMAP visualization of unbiased clustering and cell type assignment results for porcine blastoids under various parameters. k: k.param, res: resolution.

Supplementary Fig. S6 Single cell transcriptome analysis of blastoids and blastocysts. a Identification of three cell lineages based on lineage markers. **b** Pie chart showing percentage of scPred-inferred lineage cells in porcine blastoid-derived cells. Blastoid-derived cells were projected onto E7-E9 embryo dataset using scPred method. The percentages are calculated by dividing the number of cells with predicted lineage characteristics (EPI, HYPO, TE, undefined, and unassigned ones) by the total number of blastoid-derived ELCs, HLCs, TLCs, and others, respectively.

Supplementary Fig. S7 Immunofluorescent staining of IVC blastoids. a Immunofluorescent staining of IVC blastoids in iBlastoid medium on day 18. Scale bar, 200 μm. **b** Immunofluorescent staining of IVC blastoids in N2B27+AY medium on day 18. Scale bar, 200 μm.

Supplementary Table S1 Primers for real-time quantitative PCR analyses used in this study.

Gene	Forward (5'-3')	Reverse (5'-3')
EF1α	AATGCGGTGGGATCGACAAA	CACGCTCACGTTCAGCCTTT
POU5F1	TGAGGCTTTGCAGCTCAGTT	TCTCCAGGTTGCCTCTCACT
SOX2	CATCAACGGTACACTGCCTCTC	ACTCTCCTCCCATTTCCCTCTTT
NANOG	CCTACAATCCAGCTCTTTGG	CTCAGGCATTGGTGAAGATT
TBX3	TGTACATTCACCCAGACAGCC	CTTGGGAAGGCCAAAGTAAATC
TFCP2L1	TGCACGAAGAGACCTTGACC	CGCGGATGGTACTCTTCACA
KLF17	AGCACCACTTATGTGAGCCA	CTCTCACCTGTGTGTTTGCG
STAT3	CAGATCCAGTCGGTGGAACC	GTATGGGGCAGCACTACCTG
LIN28A	GTTCTGCATTGGGAGCGAGA	GGCAGTTTGCATTCCTTGGC
NODAL	CGTCTCCAGATGGACCTGTT	CTGCTCTGGAGAGAGGTTGG
OTX2	CGGAGTCCAGGGTTCAGGTA	ACTGGCCACTTGTTCCACTC
DNMT3A	TTCCTGGTATGAACAGGCCG	CTCAGTGCACCACAGGATGT

Reference

1. Zhi, M. et al. Generation and characterization of stable pig pregastrulation epiblast stem cell lines. *Cell Res* **32**, 383-400 (2022).

Supplementary Fig. S1

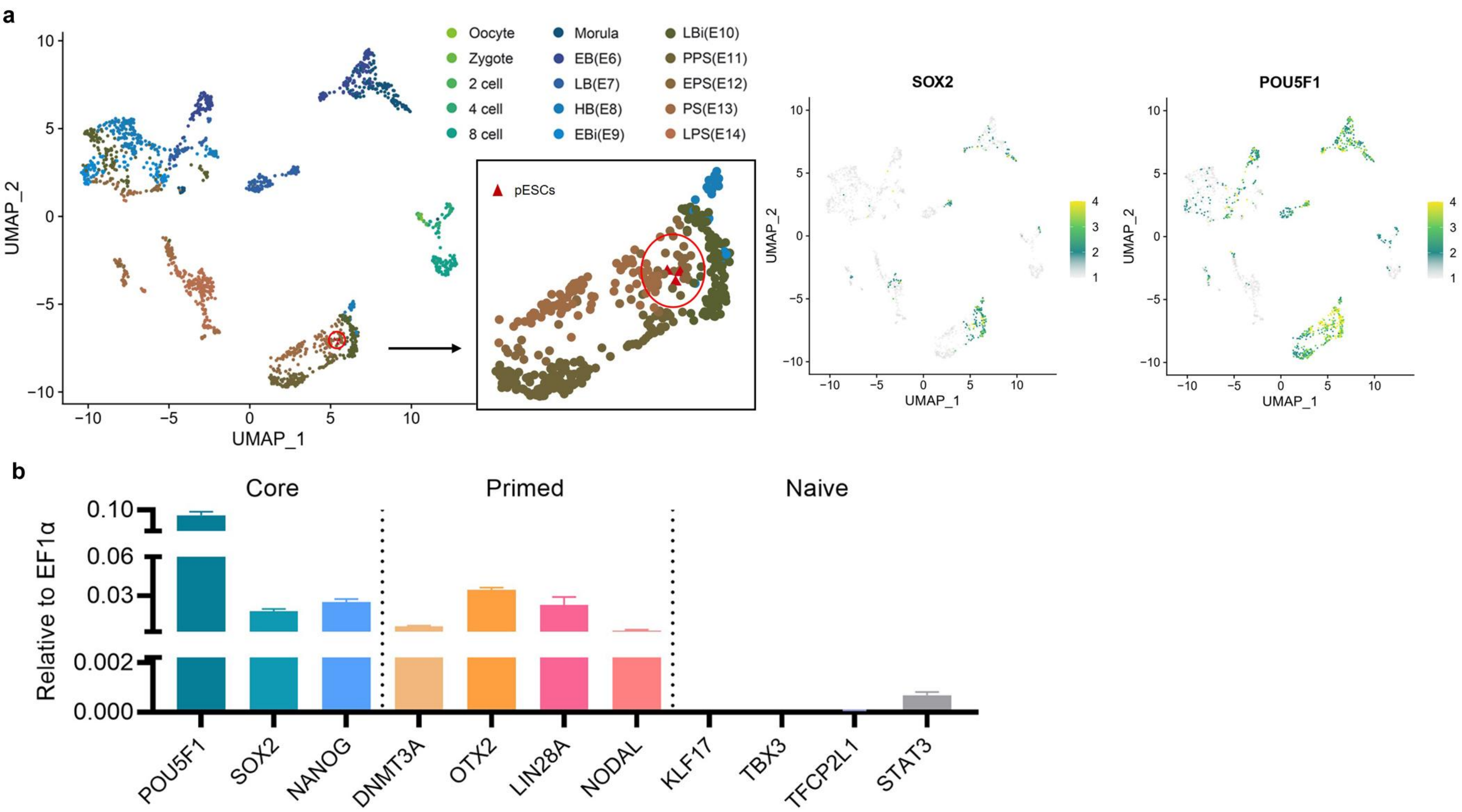

Supplementary Fig. S2

a

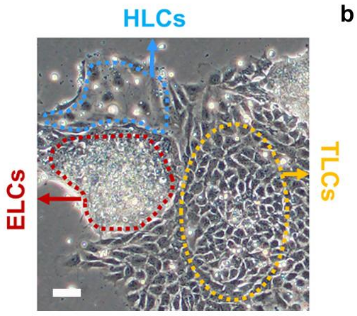

b

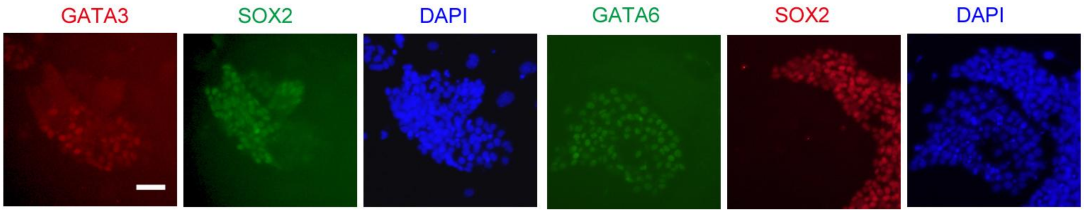

c

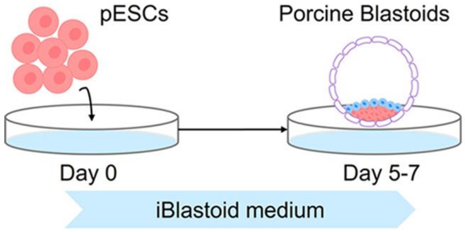

d

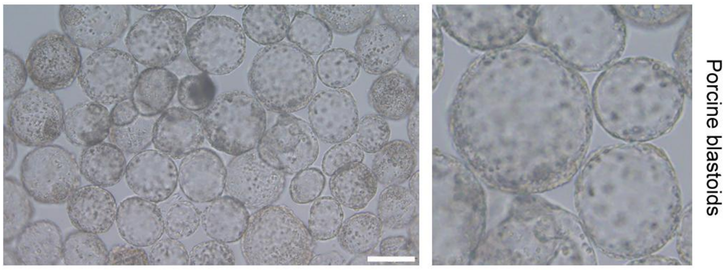

e

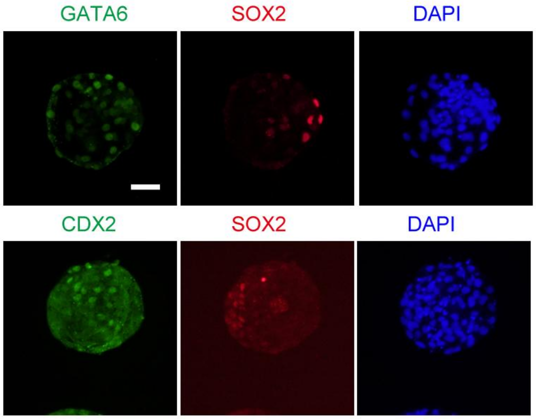

f

One-step process			Two-step process		
No. of blastoids	No. of $SOX2^+$ blastoids	Proportion of $SOX2^+$ blastoids	No. of blastoids	No. of $SOX2^+$ blastoids	Proportion of $SOX2^+$ blastoids
38	7	18.4%	50	23	46%
54	11	20.4%	74	41	55%
53	18	34%	71	36	51%
25	8	32%	119	40	33.6%
113	35	31%	81	21	25.9%

Supplementary Fig. S3

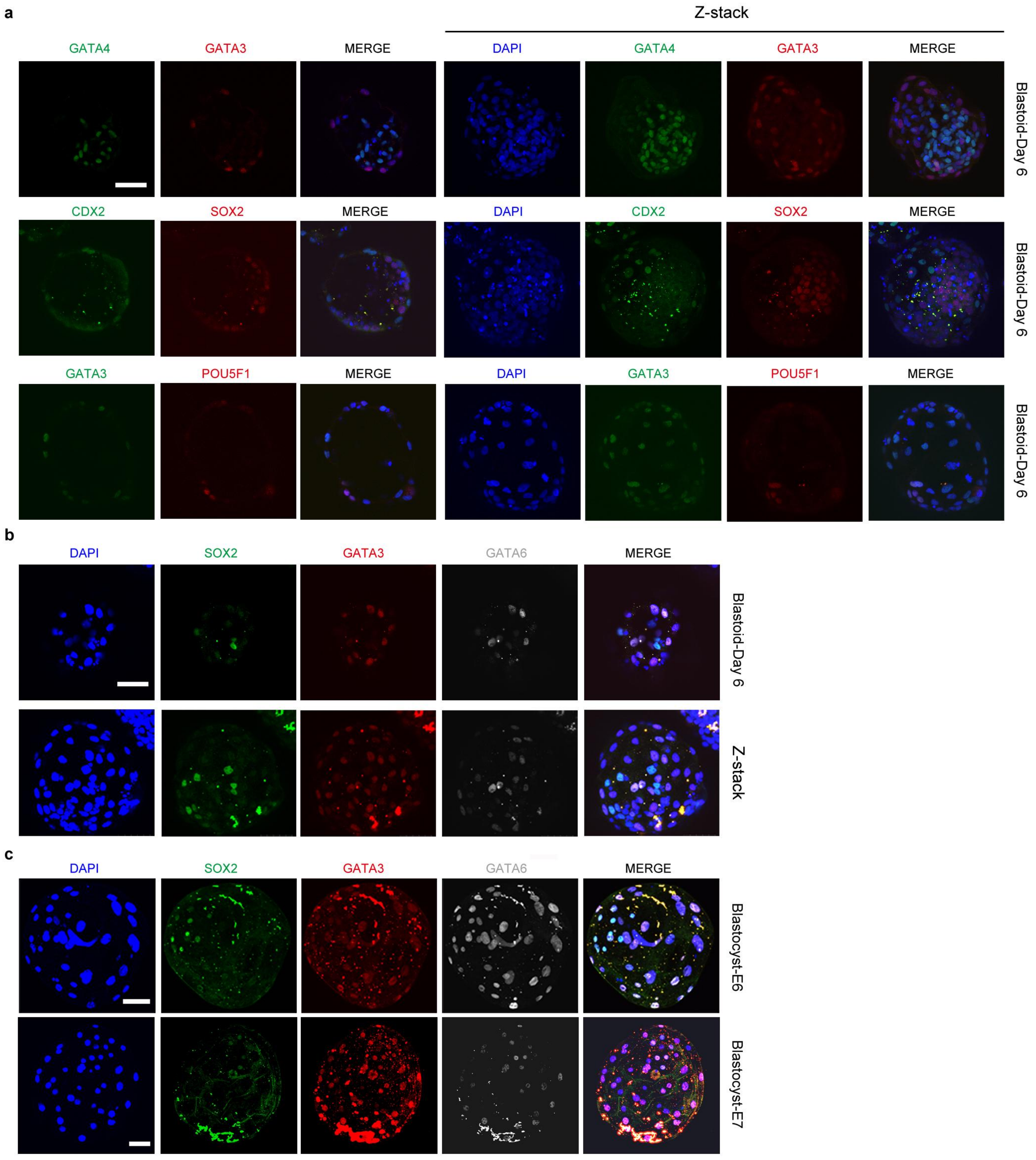

Supplementary Fig. S4

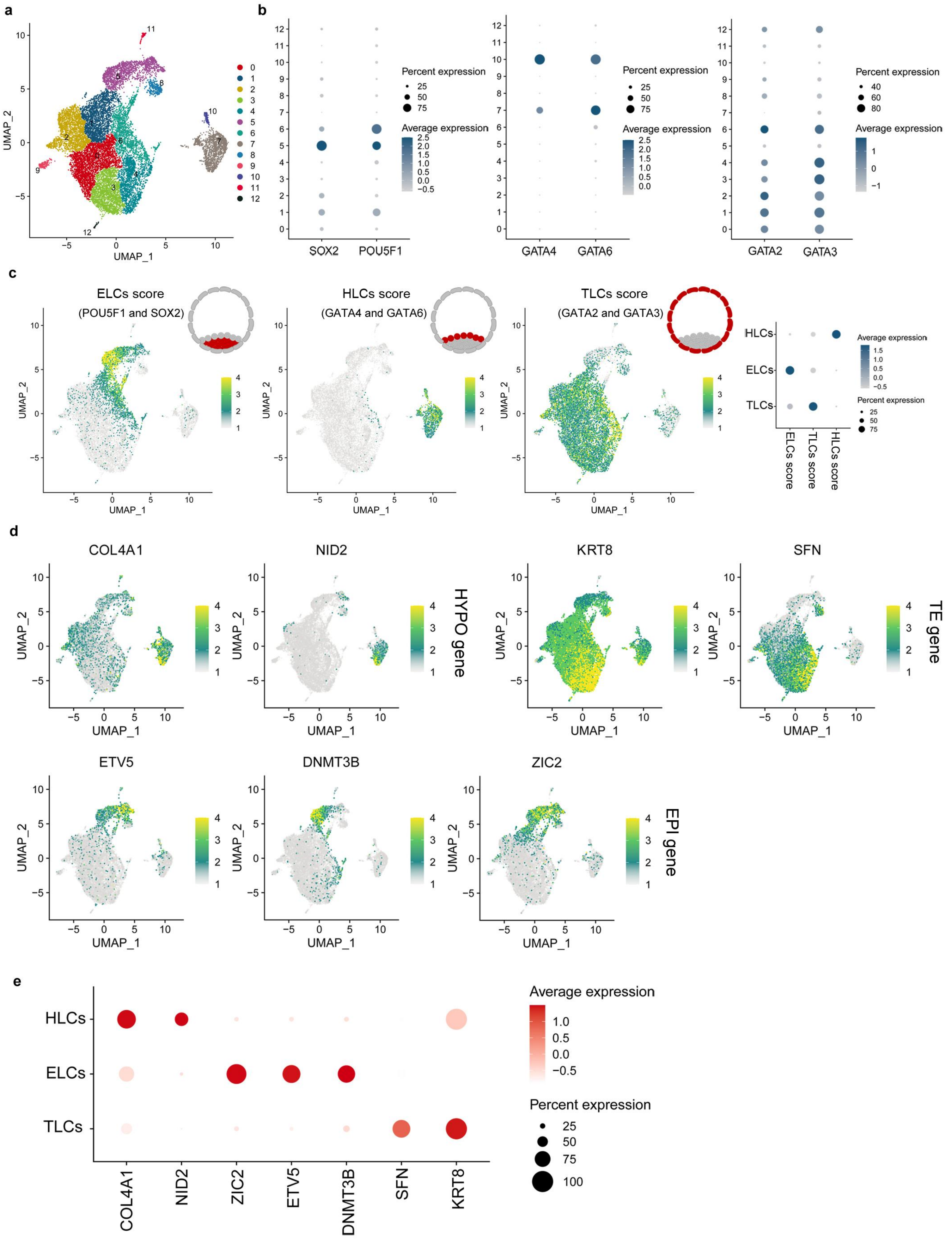

Supplementary Fig. S5

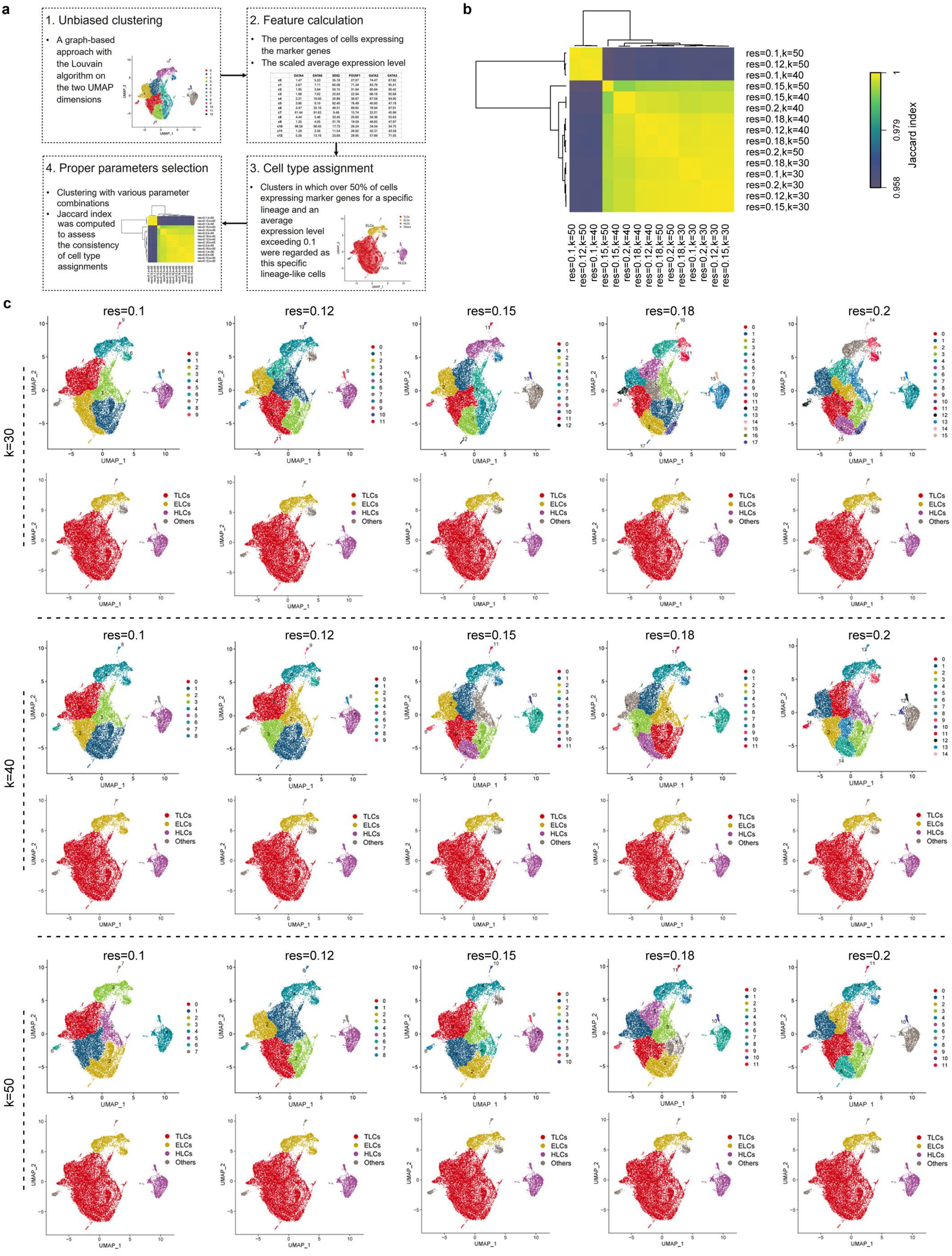

Supplementary Fig. S6

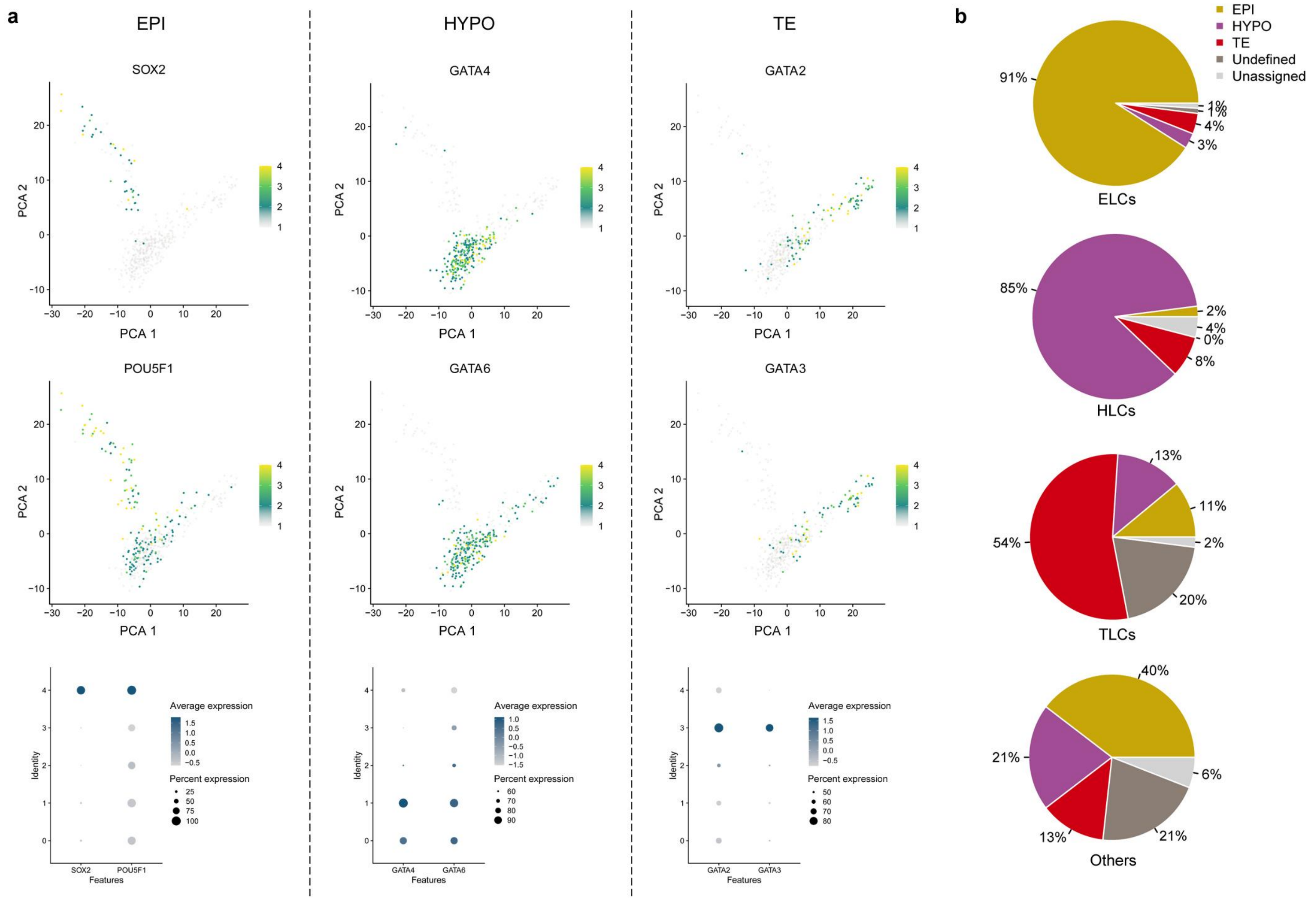

Supplementary Fig. S7

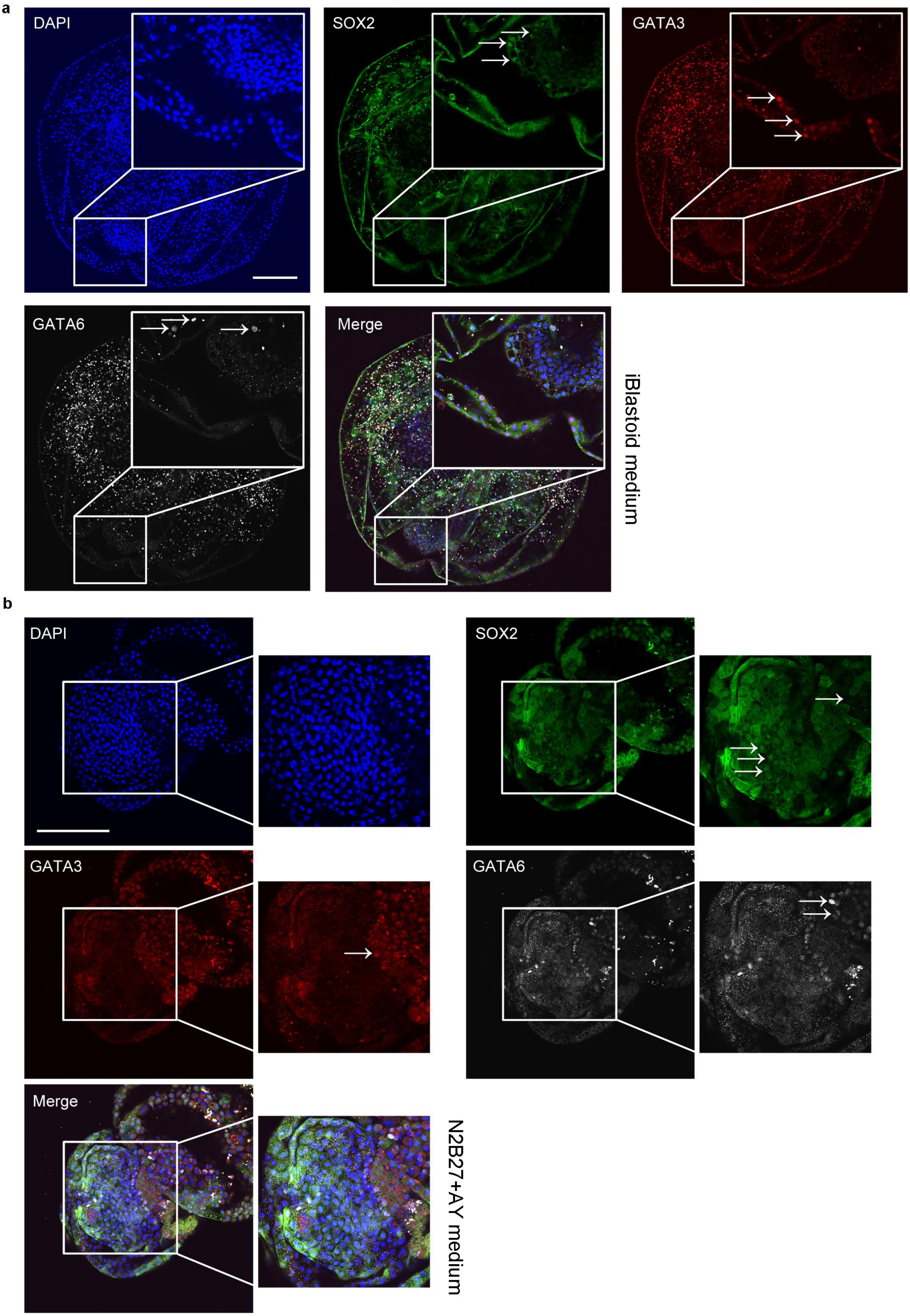

Supplement: Supplementary file 1 — Supplementary Information [file 41421_2024_693_MOESM1_ESM.pdf]
